# Posttranslational Modification of 6-phosphofructo-1-kinase as an Important Feature of Cancer Metabolism

**DOI:** 10.1371/journal.pone.0019645

**Published:** 2011-05-04

**Authors:** Andreja Šmerc, Eva Sodja, Matic Legiša

**Affiliations:** Department of Biotechnology, National Institute of Chemistry, Ljubljana, Slovenia; University of Delhi, India

## Abstract

**Background:**

Human cancers consume larger amounts of glucose compared to normal tissues with most being converted and excreted as lactate despite abundant oxygen availability (Warburg effect). The underlying higher rate of glycolysis is therefore at the root of tumor formation and growth. Normal control of glycolytic allosteric enzymes appears impaired in tumors; however, the phenomenon has not been fully resolved.

**Methodology/Principal Findings:**

In the present paper, we show evidence that the native 85-kDa 6-phosphofructo-1-kinase (PFK1), a key regulatory enzyme of glycolysis that is normally under the control of feedback inhibition, undergoes posttranslational modification. After proteolytic cleavage of the C-terminal portion of the enzyme, an active, shorter 47-kDa fragment was formed that was insensitive to citrate and ATP inhibition. In tumorigenic cell lines, only the short fragments but not the native 85-kDa PFK1 were detected by immunoblotting. Similar fragments were detected also in a tumor tissue that developed in mice after the subcutaneous infection with tumorigenic B16-F10 cells. Based on limited proteolytic digestion of the rabbit muscle PFK-M, an active citrate inhibition-resistant shorter form was obtained, indicating that a single posttranslational modification step was possible. The exact molecular masses of the active shorter PFK1 fragments were determined by inserting the truncated genes constructed from human muscle PFK1 cDNA into a *pfk* null *E. coli* strain. Two *E. coli* transformants encoding for the modified PFK1s of 45,551 Da and 47,835 Da grew in glucose medium. The insertion of modified truncated human *pfk*M genes also stimulated glucose consumption and lactate excretion in stable transfectants of non-tumorigenic human HEK cell, suggesting the important role of shorter PFK1 fragments in enhancing glycolytic flux.

**Conclusions/Significance:**

Posttranslational modification of PFK1 enzyme might be the pivotal factor of deregulated glycolytic flux in tumors that in combination with altered signaling mechanisms essentially supports fast proliferation of cancer cells.

## Introduction

A consistent characteristic of malignant cells is the consumption of a larger amount of glucose compared to that of normal cells and the conversion of the majority of glucose into lactic acid. The tumor cells preferentially use glycolysis over mitochondrial oxidative phosphorylation for glucose-dependent ATP production even in the presence of ample oxygen to fuel mitochondrial respiration. [1]. This deviant energetic metabolism, known as the “Warburg effect,” is therefore at the root of tumor formation and growth and has been even discussed as a potential hallmark of cancer [2].

In the last decade, the discovery of oncogenes diverted interest away from studies of cellular metabolism in tumors towards those aimed at uncovering the function of oncogenes that control metabolism. Thus far, the crucial factors recognized for producing the cancer metabolic phenotype appear to be the oncogenic mutations that alter growth factor signaling through the PI3K/Akt/mTOR pathway [3]. Activation of this pathway enhances metabolic activities of glycolysis by two major events. First, the synthesis of the sugar transporter Glut1 is induced to facilitate glucose uptake by the cells [4], [5]. Second, the activity of transcription complex HIF-1α is increased, which in cooperation with transcription factor c-Myc enhances the synthesis of the majority of glycolytic enzymes [6]. Increased amounts of the wild-type enzymes consequently result in increased specific activities. However, glycolytic flux in eukaryotic organisms is tightly controlled by allosteric enzymes that retain their regulation by feedback inhibition in spite of the elevated activities of intermediary enzymes. This statement has been confirmed by experiments in *E. coli* [7] and *S. cerevisiae* [8], where overexpression of all glycolytic enzymes had no effect on the rate of glucose consumption and/or ethanol production. Therefore, one is forced to conclude that important modifications of the kinetics of regulatory enzymes must also be involved in metabolic changes that occur during the transformation of normal mammalian cells into cancer cells.

Glycolysis is central to primary metabolism, and normally, it is tightly regulated by three allosteric enzymes, hexokinase, 6-phosphofructo-1-kinase (PFK1) and pyruvate kinase (PK), which catalyze individual irreversible steps. Hexokinase, involved in the first regulatory step, appears predominantly in an HK2 isoenzyme form in tumors that is bound to the mitochondrial outer membrane facing the cytosol. Microlocation of this enzyme enables preferential access to newly synthesized ATP for phosphorylating glucose, and it is resistant to product inhibition [9]. Another allosteric enzyme is pyruvate kinase, which regulates metabolic flux over the terminal part of glycolysis. Tumor cells have been shown to exclusively express the embryonic M2 isoform of PK that can be activated by fructose-1,6-bisphosphate. However, binding of tyrosine-phosphorylated peptides to PK-M2 results in the release of the allosteric activator, leading to inhibition of enzymatic activity. Deactivation of PK-M2 in tumor cells is believed to divert glucose metabolism from energy production to anabolic processes [10].

However, the most complex control over glycolytic flux is attributed to PFK1 (EC 2.7.1.11), which surmounts the regulatory roles of the other two allosteric enzymes. PFK1 catalyzes the phosphorylation of fructose-6-phosphate to fructose-1,6-bisphosphate, using MgATP as a phosphoryl donor [11]. PFK1 is stimulated by fructose-2,6-bisphosphate (F-2,6-BP), ADP/AMP and ammonium ions, whereas citrate and ATP act as strong inhibitors [11], [12].

During evolution, eukaryotic PFK1 enzymes developed by duplication, tandem fusion and divergence of catalytic and effector binding sites of a prokaryotic ancestor [12]. However, the strict conservation between active site residues in the N-terminal segment of the eukaryotic enzyme and those of bacterial PFKs suggest that the active site of eukaryotic PFK1 is located only in the N-terminal portion [12]. On the other hand, the allosteric ligand binding sites that developed during evolution by mutations in the C-terminus enable fine-tuning of the regulatory enzyme by the elevated levels of specific downstream metabolites. One of the allosteric ligands is citrate, which acts as a potent inhibitor of all mammalian PFK1 isoforms. Studies on citrate allosteric sites in rabbit muscle PFK1 concluded that these sites developed from the phosphoenolpyruvate (PEP)/ADP allosteric site of *Escherichia coli*. Amino acid residues forming the citrate allosteric site are located both in the N- and C-termini of the molecule [13].

In mammalian genomes, three different PFK1 genes are present and are differently expressed in individual tissues. In human tissues, their protein products have the following molecular masses: muscle type (PFK-M), 85,051 Da [14]; liver type (PFK-L) 84,917 Da [15]; and platelet type (PFK-P), 85,596 Da [16].

All three isoenzymes are strongly inhibited by citrate, with IC_50_ values of 0.08, 0.13 and 0.18 mM for brain (platelet), muscle, and liver PFK1, respectively [17]. All human PFK1 isoforms are also reported to be intensely inhibited by ATP concentrations higher than 0.05 mM, yet (F-2,6-BP) can antagonise the negative effects of ATP to some extent [18].

Currently, in cancer cells, the activity of the PFK1 enzymes is believed to be up-regulated only by the loss of p53 function, which results in the down-regulation of the TIGAR protein that acts as a fructose-2,6-bisphosphatase [19]. Consequently, the level of F-2,6-BP remained high in tumors and acted as a strong positive stimulus.

However, a PFK1 isoform that was less sensitive to citrate inhibition (K_i_ = 0.75 mM citrate) and more sensitive to activation by F-2,6-BP was described in human glioma [20]. A PFK1 isoform with similar kinetic characteristics was also observed in the fast-growing rodent AS-30D hepatoma cells, which showed complete insensitivity towards its allosteric inhibitors, citrate and ATP, in the presence of physiological concentrations of F-2,6-BP. In addition, the enzyme was highly activated by its activators NH_4_^+^, AMP, and F-2,6-BP [21]. Yet, the nature of PFK1 isoforms exhibiting changes in enzyme kinetics was not studied in detail.

A citrate inhibition-resistant form of PFK1 that was activated to a higher level by allosteric activators has recently been described in the commercially important fungus, *Aspergillus niger* [22]–[24]. These kinetic characteristics were attributed to 49-kDa subunits, which are relatively small PFK1 molecules with respect to other eukaryotic PFK1s of approximately 85 kDa. Further studies showed that the shorter 49-kDa fragments are formed by a two-step posttranslational modification of the native 85-kDa enzyme [23]–[25].

In the present report, we present evidence that a similar posttranslational modification of the native muscle-type PFK1 may also occur in mammalian cancer cells that consequently leads to the formation of active shorter PFK1 fragments with changed kinetic parameters.

## Results

### Analyses of amino acid sequences of the human PFK-M protein

The origin of mammalian genes encoding PFK1 enzymes by duplication of prokaryotic ancestor genes [12] can be confirmed by the alignment of amino acid residue sequences of the N- and C- halves of the human PFK-M isoenzyme, showing substantial homology (supplemental Fig. S1). Analysis conducted by CLUSTALW [26] revealed 25.4% identity, 21.6% strong similarity, 11.6% weak similarity and 41.8% difference among amino acid residues of both halves of the primary structure.

The studies on posttranslational modification of *A. niger* PFK1 showed that the native enzyme was first cleaved by serine protease to a shorter protein that was initially inactive, but regained activity after the phosphorylation of a specific threonine residue that was located in the enzyme active center [25]. A negatively charged amino acid residue (phosphorylated threonine) was essential for generating enzyme activity [25]. By replacing the codon for the threonine residue with one for glutamic acid in the truncated *A. niger pfk*A, the need for phosphorylation of initially inactive shorter PFK1 fragments was eliminated and active shorter PFK1 fragments were encoded directly by the modified *pfk*A genes [25]. By aligning the deduced amino acid sequences of three human isoenzymes with that of the *A. niger* enzyme (supplemental Fig. S2), a negatively charged amino acid residue (aspartic acid) was found only in the sequence of PFK-M at the position corresponding to the threonine residue in the *A. niger* protein. The other two isoenzymes, PFK-L and PFK-P, contained a non-polar alanine residue at the matching site. PFK-M with a negatively charged aspartic acid residue at this critical locus was therefore concluded to be the most likely candidate for generating active shorter PFK1 fragments after a single posttranslational modification step.

### *In vitro* posttranslational modification of mammalian PFK1

To verify that, PFK1 was isolated from rabbit muscle. The purified enzyme was incubated with various proteases and tested for the presence of newly generated, active, citrate inhibition-resistant shorter PFK1 fragments. Various commercially available proteases from various species were employed in individual experiments.

**Figure 1:**
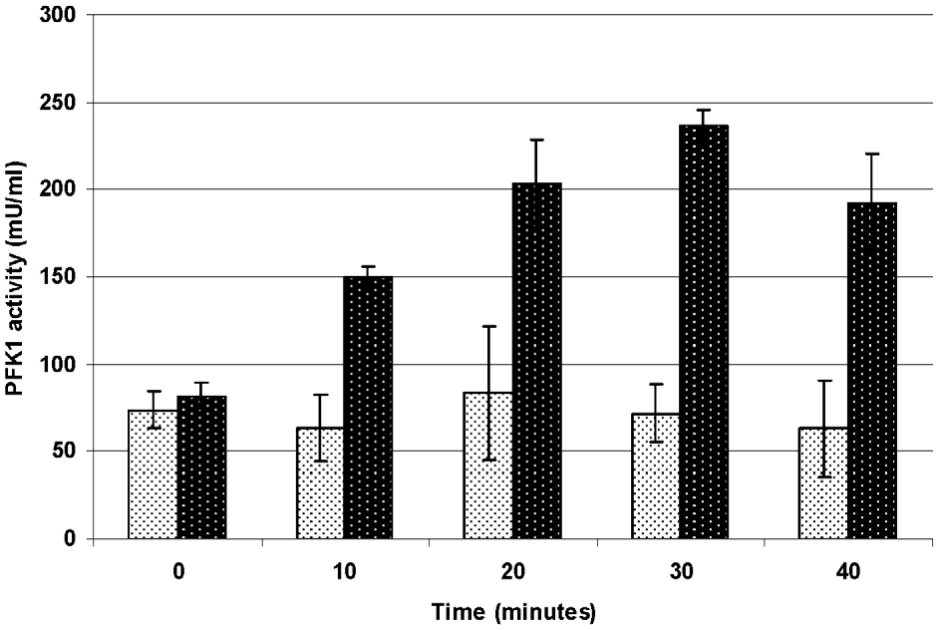
PFK1 activities after limited proteolytic degradation of native rabbit PFK1 by Proteinase K. Activities of the native PFK1 isolated from rabbit muscle after limited proteolysis by Proteinase K (dark) and untreated native enzyme (light) as measured in a system containing 5 mM citrate. Data are representative of three independent measurements and are presented as means ± standard deviation.

The experiment was conducted in a buffer containing 5 mM citrate, which functions as a strong inhibitor of the native enzyme but not of the shorter fragments. After limited proteolysis of the purified native PFK1 with Proteinase K (0.001 mg/ml), PFK1 activity was detected. A gradual increase in PFK1 activity was detected in the samples that were exposed to proteolytic action for prolonged periods of time (Fig. 1). With SDS-PAGE, fragments of approximately 45 kDa were observed after limited proteolysis with 0.001 mg/ml of Proteinase K (supplemental Fig. S3), whereas incubation with Proteinase K at a higher concentration (0.01 mg/ml) produced inactive, slightly shorter fragments. No active fragments could be detected after cleavage of the native enzyme with other commercially available enzymes of microbial or mammalian origin.

This experiment primarily showed that a single-step posttranslational modification of mammalian PFK1 was possible for yielding active shorter PFK1 fragments. The protease that was actually involved in the production of such fragments in human cells remains to be determined, but the most likely candidates are serine proteases that must be activated intracellularly.

### Detecting short PFK-M fragments in metastatic tumor cell lines by immunoblotting

To examine which PFK1 forms are present in tumor cells, four different neoplastic cell lines that are known to induce metastatic tumors after insertion into test animals were used. The following cell lines were tested: human carcinoma HeLa cells; mouse melanoma B16-F10 cells; and two lymphomas, the rat Nb2-11 line and the human TF-1 line. For western blotting, an antibody raised against an epitope of the enzyme's active center that was identical in various mammalian PFK1-M isoforms, but not in L or P isoforms, was employed.

**Figure 2:**
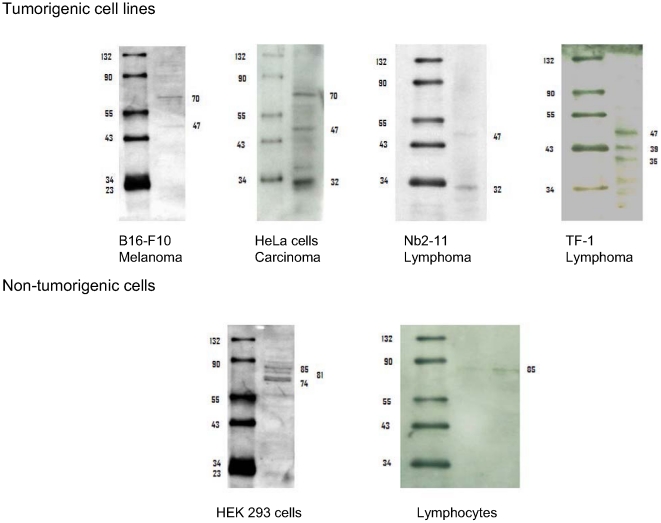
Western blots of homogenates of four tumorigenic cell lines, HEK immortalized cells and lymphocytes immunostained with PFK-M anti-bodies. Western blots of four tumorigenic cell lines (above) showed the presence of fragments of different lengths, with a fragment of 47 kDa regularly present, while no native 85-kDa PFK1 could be observed. In non-neoplastic cell lines (below) (HEK cells), the native PFK1 forms were predominant, while in normal lymphocytes isolated from peripheral human blood only a single protein band was detected corresponding to 85 kDa native PFK1. In the Western blot of lymphocytes two volumes of cell lysate were applied to the gel: 10 µl (left), and 20 µl (right).

In the homogenates of all neoplastic cell lines, the amount of native PFK1 of 85 kDa was below the immunoblotting detection limit (Fig. 2). However, a number of lower molecular weight fragments were spotted. In all cell homogenates, fragments of approximately 47 kDa were present, while some other fragments appeared sporadically. In contrast to the tumorigenic cell lines, only the native 85 kDa PFK1 enzymes were observed in lymphocytes isolated from peripheral human blood. Native enzymes were predominant also in human kidney embryonic cells (HEK 293 cell line) using an identical immunostaining method. HEK cells were immortalized by adenovirus but were not tumorigenic. Although no 47 kDa low molecular weight fragment was detected in HEK cells, some slightly shortened native enzyme forms were observed that might be a product of alternative splicing. In human muscle, an alternative transcript encoding a PFK-M isoenzyme has been reported, yielding an active enzyme with 749 amino acid residues and a molecular mass of 81,776 Da [27]. Evidence for alternative splicing of the PFK-M gene has also been reported in mice [28].

In the control, no bands were observed when a sample of growth medium was immunoblotted with the antibodies used for PFK1 detection.

### Detecting short PFK-M fragments in tumors by immunoblotting

**Figure 3:**
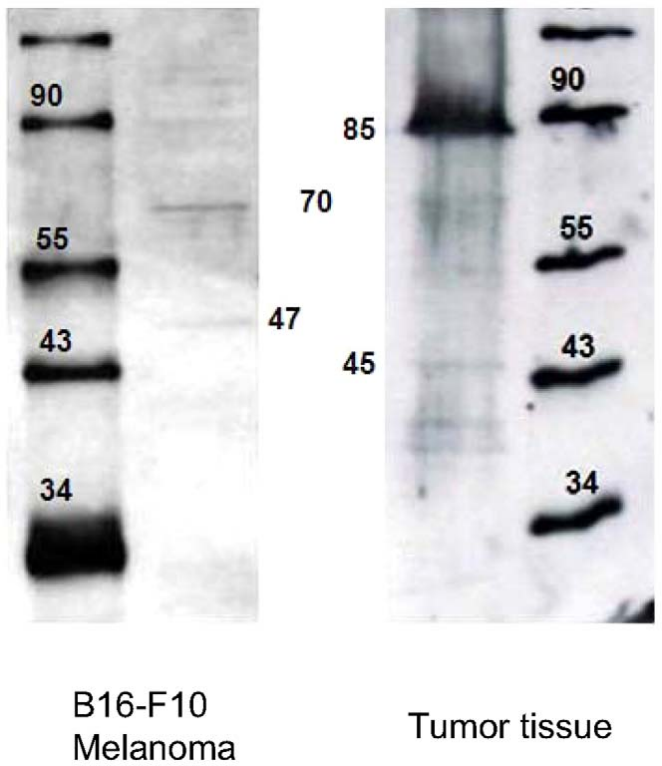
Western blots of B16-F10 cells growing as a tissue culture and B16-F10 cells that formed a tumor in mouse, immunostained with PFK-M anti-bodies. No native PFK1 enzyme was detected in the cells growing in a tissue culture, while in a tumor, a strong signal corresponding to the native enzyme was present. Shorter fragments were detected in both homogenates with a 47 fragment present in individually growing cells and a 45 kDa fragment present in a tumor tissue.

In a tumor that has developed in a C57BL/6 mouse, 10 days after the subcutaneous injection of B16-F10 cells, nearly identical fragments were detected as in B16-F10 cells growing in a tissue culture (Fig. 3). However in a tumor, a strong band corresponding to the native PFK-M enzyme was present that most probably originated from non-tumorigenic supporting tissue such as blood vessels, stroma or inflammatory cells. More detailed inspection of the shorter fragments from B16-F10 cells and corresponding tumor revealed that 47 kDa fragment was present in individually growing cells while those that developed in a tumor expressed a 45 kDa fragment.

### Truncated human muscle PFK1 cDNA encodes active shorter PFK-M fragments in *E.coli* cells with disrupted native *pfk*A

**Figure 4:**
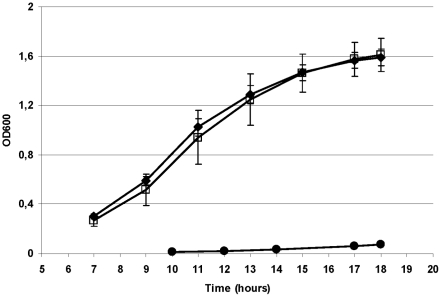
Growth of two *E.coli* transformants encoding two different human shorter PFK-M fragments. Two *E.coli* transformants encoding Fragment 4 (⧫) and Fragment 9 (□) were able to grow in supplemented glucose minimal medium. No growth of the parental strain, RL 257, carrying the pALTER-Ex-1 plasmid with no inserted gene (•) could be detected. Data are representative of three independent measurements and are presented as means ± standard deviation.

In the next step, the efficiency of active shorter human PFK-M fragments was tested in an *E. coli* strain that lacked its own native PFK1 proteins. Although the exact molecular mass of the shorter fragments could not be determined from western blots, a series of truncated genes were prepared from human muscle PFK1 cDNA. Truncated genes were inserted into the *E. coli* RL 257 [29] strain, and transformants were tested for altered growth characteristics on a medium containing glucose. The proteins encoded by truncated genes differed by several amino acid residues and covered molecular masses ranging from 45 kDa to 46 kDa and 47 kDa to 48 kDa (supplemental Table S2). Two transformants able to grow on supplemented glucose minimal medium were revealed, one from each group of molecular masses (Fig. 4). The first strain synthesized Fragment number 4 (supplemental Table S2) with 422 amino acid residues and a molecular mass of 45,551 Da, whereas the other strain encoded Fragment number 9 (supplemental Table S2) with 443 amino acid residues and a mass of 47,835 Da. The cells of both strains multiplied to an optical density of 2 in approximately 24 hours, which indicated that both recombinant proteins were active and able to effectively participate in bacterial metabolism. No growth of transformants encoding other shorter PFK-M fragments could be observed, although synthesized recombinant proteins were detected by western blots (supplemental Fig. S4). No growth on glucose medium could be detected by a control, the parental RL257 strain carrying the pALTER-Ex1 plasmid with no gene inserted. Surprisingly, the transformant encoding the native human PFK-M (85,051 Da) was unable to proliferate under identical conditions, although high enzymatic activity (more than 600 mU/ml) was detected in the cell-free extract.

**Figure 5:**
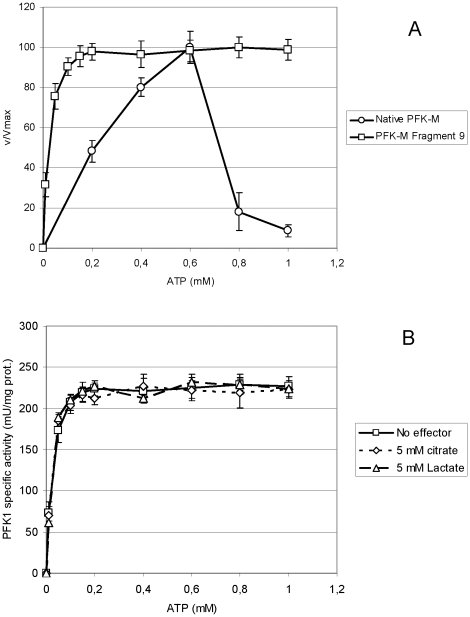
PFK1 activity of a recombinant shorter PFK-M fragment and native PFK-M enzyme with respect to some inhibitors. In figure 5A relative specific PFK1 activities detected in the homogenate of the transformant encoding Fragment 9 (□ ) and with native PFK-M enzyme isolated from *E.coli* transformant (○) are shown, that were measured at increasing concentrations of ATP. In figure 5B specific PFK1 activities were measured in the homogenate of the transformant encoding Fragment 9 without inhibitor (□), in the presence of 5 mM Na_3_-citrate (◊), and with 5 mM Na-lactate (Δ). All measurements were conducted with 0.5 mM of F6P. Data are representative of at least three independent measurements and are presented as means ± standard deviation.

In both transformants that were able to grow on glucose medium, PFK1 activity was detected in the homogenates. In both transformants that were able to grow on glucose medium, PFK1 activities were detected in the homogenates. In the transformant encoding Fragment 9, activity resistant to ATP and citrate inhibition was recorded at 0,5 mM of F6P which is near physiological concentration [30]. The Fragment 9 showed high affinity toward the ATP (K_m_ of about 0.05 mM) while at concentrations higher than 0.2 mM, no ATP inhibition could be detected (Fig. 5A). On the contrary, the recombinant human native 85 kDa PFK-M isolated from *E.coli* RL257 strain showed a peak in the enzyme activities at increasing concentrations of ATP. At low ATP concentrations the activities rose more slowly in respect to the shorter fragment, indicating lower affinity of the native enzyme toward the ATP (K_m_∼0,3 mM). However, ATP concentrations above 0,6 mM caused a sharp decrease of the native enzyme activity and only a modest PFK1 activity was detected at ATP concentration of 1 mM (Fig. 5A). Sodium citrate didn't inhibit the activity of the shorter PFK-M fragment (Fig. 5B). This is in contrast to the kinetic characteristics of the recombinant human native PFK-M enzyme where a strong sensitivity toward the citrate was revealed [31]. No inhibition of the shorter fragment with lactate could be detected either (Fig. 5B), a metabolite that was recently proposed to down regulate mouse PFK1 activities [32].

**Figure 6:**
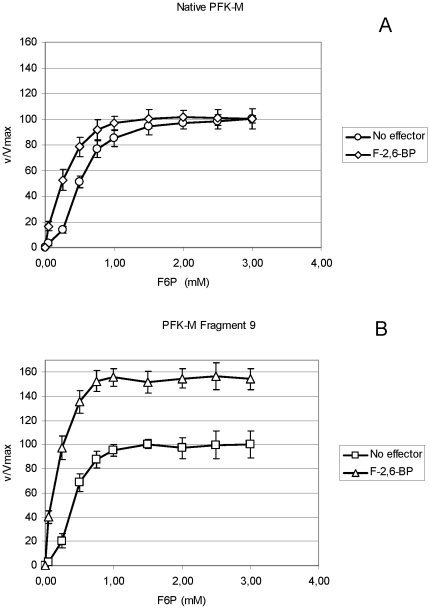
PFK1 activity of a recombinant shorter PFK-M fragment and native PFK-M enzyme with respect to F-2,6-BP as an activator. In figure 6A F6P saturation curves of the isolated native PFK-M enzyme with (○) and without (◊) 4 µM of F-2,6-BP are presented. In figure 6B F6P saturation curves detected in the homogenate of the transformant encoding Fragment 9 with (□) and without (Δ) 4 µM of F-2,6-BP are shown. The measurements were conducted with 1 mM of ATP. In both graphs relative specific activities are shown. Data are representative of at least three independent measurements and are presented as means ± standard deviation.

Fructose-6-phosphate saturation curves without and with F-2,6-BP showed a change in PFK-M activities of both the native enzyme (Fig. 6A) and the Fragment 9 (Fig. 6B). By adding F-2,6-BP to the measuring system, a sigmoid plot was converted to Michaelis-Menten kinetics, characterized by a steep rise in activities with respect to substrate concentration. Although F-2,6-BP increased the affinity of both enzymes toward the F6P as a substrate, the activator also caused a marked increase in maximal velocity of the shorter Fragment 9 (Fig. 6B) while no such effect could be recorded with the native enzyme (Fig. 6A).

The shorter PFK-M fragments appeared to be extremely unstable in the in vitro conditions. The activity could be stabilized to a certain extent in a cell-free extract that contained about 10 mg of proteins per ml by adding fructose-6-phosphate to a final concentration of 6 mM. However, rapid deactivation was recorded in the measuring vial (supplemental Fig. S5) when the amount of dissolved proteins was significantly reduced. After approximately 10 minutes of incubation at 30°C, no NADH consumption could be detected in the system.

### Expression of h*pfk*MFrg9 in non-tumorigenic HEK 293 cells promotes growth, glucose consumption and lactate production

**Figure 7:**
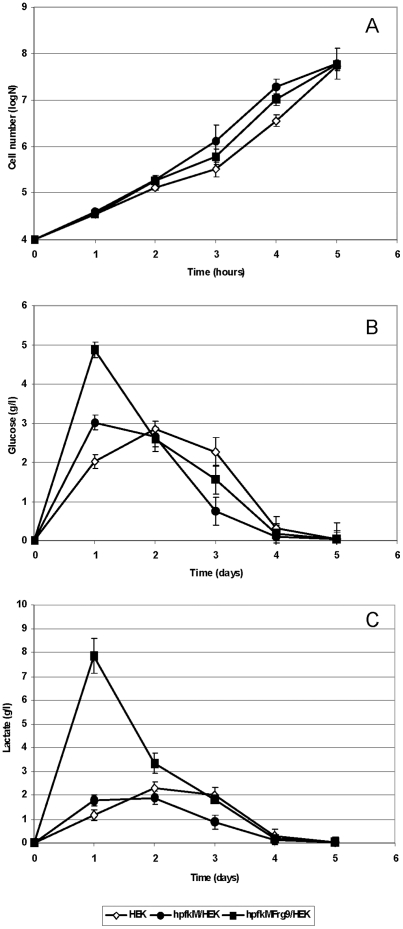
Growth, glucose consumption and lactate excretion by the stably transfected HEK cells synthesizing PFK1 Fragment 9, the native PFK-M and HEK cells with empty plasmid. In figure 7A growth of Flp-In T-Rex HEK 293 cells with integrated h*pfk*MFrg9 (h*pfk*MFrg9/HEK - ▪) encoding the Fragment 9; integrated h*pfk*M (h*pfk*M/HEK - •) encoding the native PFK-M enzyme; and cells with integrated empty plasmid (HEK - ◊) are presented in logarithmic mode. In figure 7B glucose consumption by transfectants normalized to 1 million cells is shown. In figure 7C lactate production recalculated to 1 million cells is presented. Identical symbols for individual transfectants are used in all figures. Data are representative of three independent measurements and are presented as means ± standard deviation.

To determine whether modified PFK-M enzymes have similar physiological effects in non-tumorigenic human cells (Flp-in T-Rex HEK 293 cell line), stable transfectants were prepared that enabled constitutive expression of h*pfk*M encoding the native PFK-M and h*pfk*MFrg9 encoding the PFK-M Fragment 9. Growth rate, glucose consumption and lactate accumulation were compared with those in transfectants carrying integrated empty plasmid under identical growth conditions. The cells expressing h*pfk*M and h*pfk*MFrg9 proliferated more rapidly compared to the parental cells, as observed on semi-logarithmic graph, however detailed analyses of a linear plot of the same data suggested a slightly shorter lag phase of transfected cells with respect to the parental strain (Fig. 7A). The most drastic difference among tested transfectants was observed for lactate excretion. At 24 hours of incubation, the amount of lactate accumulated in the medium and normalized to 1 million cells revealed four folds higher productivity by the strain synthesizing Fragment 9 with respect to the strain encoding the native PFK-M and six folds higher in comparison to the parental strain. At day two, the amount of lactate accumulated was still about 30% higher by the cells with Fragment 9, whereas later, similar values were obtained by all three tested cell lines (Fig. 7C). Increased lactate production by the cells expressing the h*pfk*MFrg9 gene was reflected also in the glucose consumption rates. At 24 hours, the highest amount of glucose, normalized to the fixed cell number, has been taken up by the cells encoding the Fragment 9, about 40% less glucose was consumed by the cells synthesizing the native PFK-M enzyme, while the parental cells metabolized even less glucose (Fig. 7B).

## Discussion

A variety of oncogenes, including Akt [33], BCR-Abl [34], c-Myc and HIF [35], promote glucose metabolism in cancer cells. However, the activation of Akt alone, which encodes a serine/threonine kinase that is under the control of phosphatidylinositide-3-kinase PI3K, has been proven sufficient to stimulate the switch to aerobic glycolysis [36]. However, the underlying molecular changes at the level of regulatory glycolytic enzymes remain poorly understood. Constitutive activation of Akt has been implicated in the regulation of cell proliferation [37] and suggested to participate in promoting Glut1 transporter activity [4]. Moreover, the stimulating role of the PI3K/Akt signaling pathway has been reported in hormone-dependent proteolytic induction (kallikrein gene expression) in breast [38] and prostate [39] cancer cell lines. Human tissue kallikreins belong to a subgroup of serine proteases that are similar to Proteinase K, which we have demonstrated here to cleave native PFK1 enzymes to form active, citrate inhibition-resistant shorter PFK1 fragments. Therefore, Akt-mediated induction of aerobic glycolysis might also be involved in the posttranslational modification of PFK1 by activating proteolytic enzymes.

This assumption is supported by the similar results obtained with transfected cells constitutively expressing Akt [36] and the cells synthesizing highly active shorter PFK1 fragments in this study. Both types of cells consumed glucose more rapidly and excreted lactate in higher yields compared to the un-transfected cells.

With Western blotting experiments of tumorigenic and normal cells no native 85 kDa enzyme could be detected in neoplastic cells, whereas a fragment of 47 kDa was characteristically present. However, some other smaller fragments were spotted as well. They most probably originated from the native PFK1 since the antibody used, proved to be specific enough and no low molecular peptides appeared in the lysate of lymphocytes and HEK cells. Besides, little is known about the cytosolic proteolytic activity in cancer cells therefore it is difficult to speculate about the number of proteases that might attack the PFK1 enzyme. Undoubtedly, better information about the posttranslational modification would be achieved by using an epitope-tagged PFK1 allele. In fact we tested AU1 epitope tag [40] that was N-terminally fused to the native PFK-M. Although tagged *pfk*-M gene was expressed and protein detected, no enzyme activity could be detected (data not shown). We believe that the extension of six amino acid residues influenced the folding of the protein in the cells and prevented the correct association of monomeres into an active tertameric holoenzyme. Unfortunately, inactive enzyme could not be employed for the studies of posttranslational modification by proteolytic cleavage.

Interestingly, slightly different shorter fragments were detected in B16-F10 cells growing individually and in a tumor tissue. Though, in vivo experiments conducted in *E.coli* transformants revealed that both 45 and 47 kDa PFK-M fragments could associate into an active holoenzyme. This observation suggested that different environmental conditions might influence the posttranslational modification of PFK-M in B16-F10 cells.

After posttranslational modification of PFK-M, enzyme activity is preserved, since the active site of the eukaryotic PFK1s is located in the N-terminus [12]. However, kinetic characteristics of the shorter PFK-M fragments are changed (Fig. 5–6). Most important modified enzymes become insensitive to citrate and ATP inhibition. By a proteolytic cleavage of the C-terminal part of the native molecule some components of the citrate binding site are lost, as well as a motif responsible for the inhibition by ATP (supplemental Fig. S1). Similar kinetic changes of the modified PFK1 fragments were also observed after the posttranslational modification of the native PFK1 enzyme in the filamentous fungus *Aspergillus niger* [24]. Isolated, highly active shorter fragments were resistant to citrate and ATP inhibition, while F-2,6-BP significantly increased the activities of the shorter fragments but not of the native PFK1 protein.

All kinetic measurements for human shorter PFK1 fragments were thus far conducted only in a crude enzyme preparation. It appeared that cleavage of the C-terminal segments of the holoenzyme, which is known to stabilize the tetrameric quaternary structure of the native eukaryotic protein [41], made the holoenzyme more susceptible to dissociation. In fact, according to the recently published crystal structure of rabbit skeletal muscle PFK-M, distal parts of C-teminus are responsible for the formation of tetrameric holoenzymes. Therefore, it seems very likely that the shorter fragments, lacking major part of C-terminus can assemble only in dimmeric forms [42]. Inactivation of PFK1 by dissociation at low protein concentrations was previously reported and well characterized for the rat liver enzyme [43]. The extreme instability of PFK1 appeared to be the major reason that the phenomenon of posttranslational modification was overlooked until recently.

The efficiency of shorter human PFK1 fragments was also assessed under in vivo conditions. Interestingly, only fragments with precise amino acid residue numbers were able to fold into active holoenzymes in *E. coli*. Extending the protein chain by one or two amino acid residues resulted in a complete loss of activity. These data suggested the need for a specific protease that cleave at a specific target. Similarly, the accurate length of the shorter fragments that allowed for enzyme activity was also recorded for shorter forms of *A. niger* PFK1 [25]. *E. coli* transformants that grew on glucose carried truncated genes of two different lengths and encoded PFK-M subunits of 45,920 Da (422 amino acid residues) and 47,825 Da (443 amono acid residues). In fact, these subunits were the relative molecular masses of both active fragments that were formed after limited proteolytic digestion of the rabbit PFK-M and the fragments detected by western blots.

The effect of shorter PFK-M fragments on metabolism was confirmed by an in vivo test also in mammalian cells. Although accelerated glucose consumption and lactate production was detected only at the early time points by the transfectant encoding the shorter PFK-M fragments, the data suggested the important role of the modified human PFK-M on de-regulated metabolic flux through glycolysis. However, no significant increase in growth rate among various transfectants was observed. It is important to realize that HEK 293 cell are non-tumorigenic, therefore no corrupted signal transduction by oncogenic mutations via the PI3K/Akt/mTOR pathway was present which supported fast cellular biosynthesis in cancer cells. The decline in glucose consumption and lactate excretion rate that prevailed after the first day of incubation of the transfectant encoding Fragment 9, could be triggered by lactate itself. By comparing metabolic pathways between cancer cells excreting lactate and non-tumorigenic stroma cells in colorectal carcinomas [44], it has been revealed that tumor associated fibroblasts highly expressed MCT1/2 protein which could capture lactate released from cancer cells. Stroma cells expressed also high levels of pyruvate dehydogenase (PDH) that regulated the step from pyruvate to acetyl CoA, and enable further oxidation of pyruvate. Initially induced lactate overflow detected in HEK 293 transfectants encoding the shorter PFK-M fragments (Fig. 6C) might have triggered similar metabolic changes that were reflected in a transient increase in lactate excretion.

As previously reported, the insertion of modified *pfk*A genes encoding highly active, citrate inhibition-resistant shorter PFK1 fragments enhanced the production of extracellular metabolites in fungal cells. *A. niger* cells with integrated modified *pfk*A genes showed substantially accelerated synthesis of citric acid [25], whereas *Aspergillus terreus* transformants exhibited faster excretion of itaconic acid [45]. Both carboxylic acids are primary metabolites that appear to be transported out of cells to balance anaplerotic and cataplerotic reactions in cells under the conditions of deregulated glycolytic flux.

Posttranslational modification of PFK1 enzyme might exhibit another important role in cancer metabolism. Tumors were characterized also by the exclusive production of alternatively spliced M2 form of pyruvate kinase (PKM2). PKM2 differs from PFK1 in that its activity could be negatively regulated in response to growth factor signaling by binding to tyrosine-phosphorylated proteins [10]. However, the concentrations above 20 µM of fructose-1,6-bisphosphate (FBP) were able to compete for binding of recombinant PKM2 to phosphotyrosine peptides and prevented the inhibition [10]. Even more, FBP has been also shown to act as a strong stimulator of PKM2 activity [46]. It is hard to believe that PKM2 was inhibited in tumor cells since about 90% of total glucose metabolism was accounted for lactate and alanine production in glioblastoma, therefore undisturbed metabolic flux through overall glycolysis was necessary [47]. By investigating the changes of metabolite concentrations during the progression of normal mouse mammary epithelial cells to an isogenic series of breast tumor cell lines with increasing metastatic potentials, several glycolytic intermediates were found to be substantially increased in tumorigenic lines [48]. One of them was FBP, a product of PFK1 catalytic activity, which concentration was nearly seven fold higher in the most metastatic cell line (4T1) than in a normal murine mammary gland epithelial cell line (NMuMG). Such increase of FBP intracellular concentration could be easily caused by a lack of feed back inhibition at the level of PFK1, due to the posttranslational modification of the enzyme.

In conclusion, posttranslational modification of PFK1 might trigger the most important change in the regulation of glycolytic flux in cancer cells and might have an important impact on the Warburg effect. There are indications that the PI3K/Akt signaling pathway might be involved in the process by activating a specific proteolytic enzyme that conducts the modifications.

Due to the probable confinement of the shorter PFK1 fragments to cancer cells, they could serve as useful markers for rapidly growing malignant cells. Because the primary role of the glycolytic pathway in proliferating cells is believed to be the synthesis of precursors for cellular building blocks (amino acids, nucleotides, lipids) [3], the short PFK1 fragments may also become important targets for uncoupling the synthetic and energetic pathways in cancer cells.

## Materials and Methods

Additional procedures are discussed in Supplementary Data (Text S1).

### Limited proteolytic degradation of rabbit PFK1

Aliquots of 20 µl of purified PFK1 from rabbit skeletal muscle in a phosphate buffer (pH 7.8) were incubated at 30°C with 1 µl of Proteinase K (Sigma-Aldrich, Steinheim, Germany) to give a final concentration of 0.001 mg/ml. After predetermined incubation periods, proteolytic activity was blocked by adding 1 µl of PMSF, a serine protease inhibitor, to reach a final concentration of 1 mM. Total reaction mixture (22 µl) was then transferred into the measuring system with 5 mM of citrate and the enzyme activity recorded by an assay, as described under the supplementary data (Text S1). As a control, purified rabbit PFK1 was incubated under the identical conditions without proteolytic enzyme. In addition to Proteinase K, other proteolytic enzymes were tested at the following concentrations: Furin, 0.3 to 3.2 U/ml; Cathepsin C, 1 to 10 U/ml; Cathepsin B, 1 to 10 U/ml; Urokinase 1, to 10 U/ml; and Subtilisin, 0.0001 to 0.1 mg/ml. All proteolytic enzymes were purchased from Sigma-Aldrich (Steinheim, Germany).

### Immunoblotting

Polyclonal rabbit antibody was raised by the GenScript Corporation (Piscataway, NJ, USA; www.genscript.com) against the specific epitope (CKDFREREGRLRAA) and purified by affinity chromatography. This sequence is characteristic of only mammalian PFK-M enzymes but not the PFK-P and PFK-L isoforms. Moreover, the search for epitope similarity by BLASTP (NCBI) [49] revealed that such sequence is confined exclusively to eukaryotic PFK1 enzymes.

For western blotting, samples of cell line homogenates containing approximately 20 µg of protein were separated by SDS-PAGE using 10% polyacrylamide gels with 0.1% sodium dodecyl sulfate. The transfer of proteins to a nitrocellulose membrane was confirmed by Ponceau Red. The membrane was blocked with I-Block reagent (Tropix Inc., Bedford, MA), washed, and incubated first with a 1∶700 dilution of purified primary antibodies (rabbit polyclonal IgG) and subsequently with a 1∶2000 dilution of goat anti-rabbit-HRP secondary antibodies (Abcam, Cambridge, UK). The membrane was developed with Ilford PQ Universal paper developer (Harman Technology Ltd., Mobberley, UK). Molecular mass standards (Santa Cruz Biotechnologies, Santa Cruz, CA) were used in order to determine the molecular masses of the PFK fragments. No bands were observed after a sample of growth medium was immunoblotted with the antibodies used for PFK1 detection.

### DNA manipulation

DNA manipulations were essentially done as described by Sambrook and Russel [50]. PCR reactions were performed with Platinum® *Pfx* DNA polymerase (Invitrogen, Carlsbad, CA) using the reaction solution recommended by the manufacturer. DNA was sequenced by MWG-Biotech AG (Ebersberg, Germany).

### Construction of truncated *pfk*M genes from human *pfk*-M cDNA

Human muscle-type PFK1 cDNA (Clone ID2964710) was purchased from Geneservice Ltd (www.Geneservice.co.uk). Native human *pfk*-M was amplified by PCR using 5′-AATTATGGATCCATGACCCATGAAGAGCACC-3′ as a forward primer and 5′-AATTATTCTAGATTAGACGGCCGCTTCCCC-3′ as a reverse primer. Simultaneously, restriction sites were introduced at the 5′ (*Bam*HI) and 3′ (*Xba*I) ends that enabled cloning into the pALTER-Ex1 plasmid (Promega, Southampton, UK).

For the construction of truncated human *pfk*-M genes, *Bam*HI/*Xba*I fragments of nine different lengths were prepared by PCR reactions using the oligonucleotides listed in the table S1 and then sub-cloned into the pALTER-Ex1 plasmid under the control of the *tac* promoter. Finally, the correct nucleotide sequences of the native gene and all truncated genes were verified.

### Transfection of h*pfk*MFrg9 into non-tumorigenic HEK 293 cells

Fragment 9, encoded by h*pfk*MFrg9, was cloned into the pcDNA5/FRT/V5-His TOPO plasmid (Invitrogen, Carlsbad, CA) by standard PCR-based strategies and confirmed by sequencing. Transfection of Flp-In T-Rex-HEK293 cells was performed with Lipofectamine 2000 (Life Technologies, Gaithersburg, MD). For co-transfection, the pcDNA5/FRT/V5-His TOPO plasmid with inserted h*pfk*MFrg9 and the pOG44 plasmid (Invitrogen, Carlsbad, CA) constitutively expressing Flp recombinase were used. Hygromycin was used as a selective marker at a concentration of 200 µg/ml.

Un-transfected parental cells were grown in high glucose DMEM, 10% FBS medium with 100 µg/ml of zeocin and 20 µg/ml of blasticidin. Stably transfected cells with inserted h*pfk*MFrg9 were grown in identical medium; however, zeocin was replaced by 200 µg/ml of hygromycin. As a control, parental strain transfected with the plasmid without the inserted h*pfk*MFrg9 gene has been taken.

## Supporting Information

Figure S1 **Alignment of N- and C- termini of human PFK-M.** Substantial homology can be found among N- and C-terminus of human PFK-M. Markers below the amino acid sequence represent: *, identity; : , strongly similarity; ., weakly similarity. Amino acid residues shown by white letters on black basis present citrate allosteric binding site [12]. Amino acid residues at the C-terminus extension marked with grey background represent the motif responsible for inhibition by ATP [13].
(TIF)

Figure S2 **Alignment of the deduced amino acid residues of the PFK1 active center of human PFK1 proteins PFK-M (P08237), PFK-P (Q01813), PFK-L (P17858), and** ***A. niger*** **(P78985) are shown.** The threonine (T) residue, located in the enzyme active center, must be phosphorylated in order to regain activity of the shorter *A. niger* PFK1 fragmen [14]. Only the aspartate residue (D) of PFK-M exhibits a negative charge similar to the phosphorylated threonine in the *A. niger* fragment, unlike the alanine residues of the PFK-P and PFK-L human isoforms (while letters). Allosteric binding sites for ATP are marked with a gray background.
(TIF)

Figure S3 **SDS-PAGE of the native PFK1 after limited proteolytic degradation by Proteinase K.** SDS-PAGE of the native PFK1 isolated from rabbit muscle revealed the formation of a 45-kDa fragment after limited proteolysis by Proteinase K. From left to right: purified native PFK1 from rabbit muscle; Proteinase K; native PFK1 after limited proteolysis with Proteinase K (0,001 mg/ml).
(TIF)

Figure S4 **Western blot of PFK-M fragments synthesized in** ***E.coli*** **.** Fragments of various length were encoded by a series of truncated *pfk*M genes in *E.coli* strain RL257 lacking its own PFK1 enzymes.
(TIF)

Figure S5 **Instability of the shorter PFK-M fragment under in vitro conditions.** PFK1 activity was rapidly lost in the measuring system with low protein concentration. Data are presented as means ± standard deviation.
(TIF)

Table S1 **Primers used for the construction of truncated human** ***pfk*** **genes.** Reverse primers used for the construction of truncated genes of different lengths from human muscle PFK1 cDNA by PCR reaction are shown. In all reactions, the following oligonucleotide was used as a forward primer: 5′-AATTATGGATCCATGACCCATGAAGAGCACC-3′.
(TIF)

Table S2 **Shorter human PFK-M fragments tested in** ***E.coli*** **.** Fragments of specified molecular masses and amino acid residues encoded by different truncated genes are shown in columns 1 to 3. Identical fragments would be hypothetically formed after proteolytic cleavage of the native human PFK1 (type M) at the specified target sequences (column 4).
(TIF)

Text S1 **Supplementary data**
(DOC)
