# Supplementary figures and images for: Posttranslational Modification of 6-phosphofructo-1-kinase as an Important Feature of Cancer Metabolism
Source: PLoS One. 2011 May 4;6(5):e19645. doi: 10.1371/journal.pone.0019645 (PMC3087806; doi:10.1371/journal.pone.0019645)

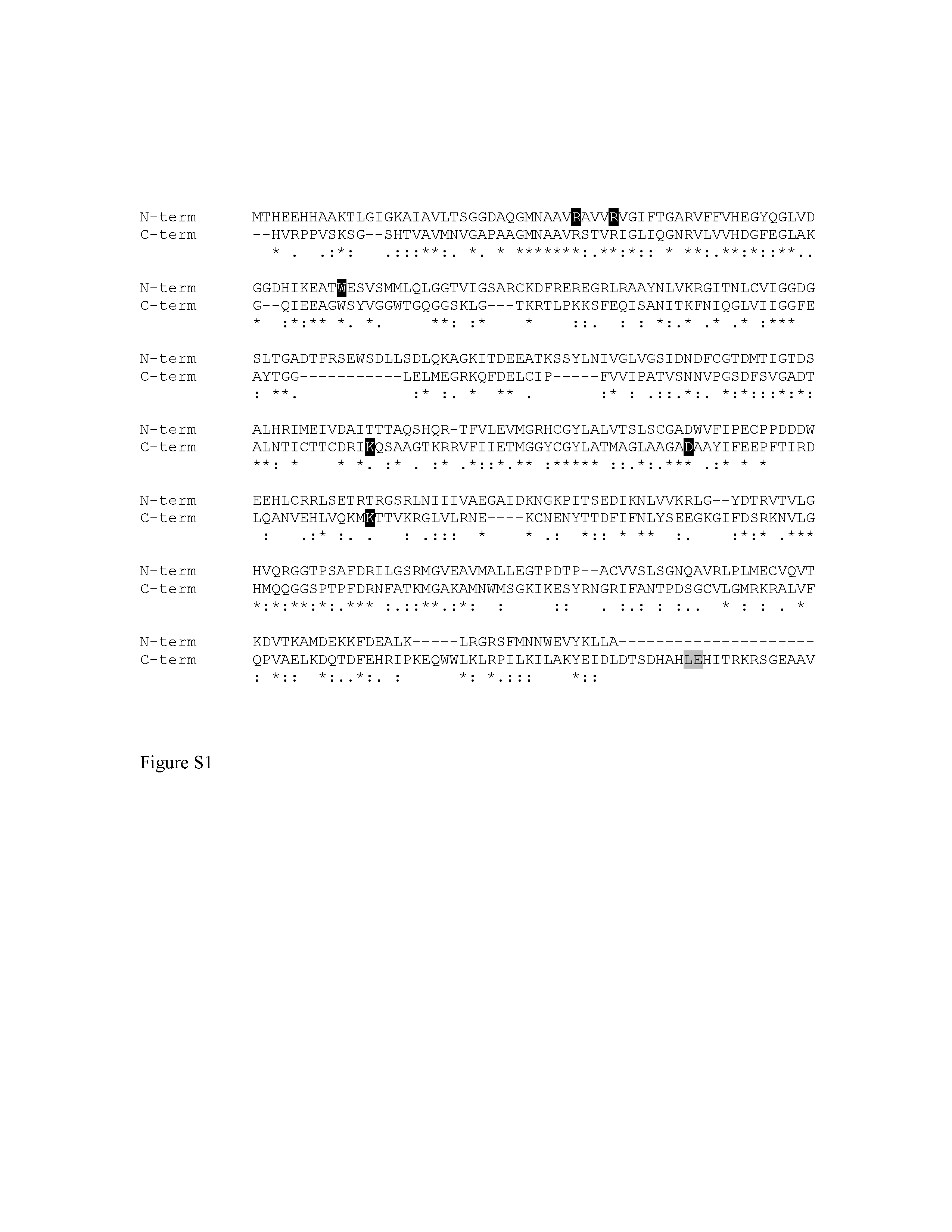

Supplement: Figure S1 — Alignment of N- and C- termini of human PFK-M. Substantial homology can be found among N- and C-terminus of human PFK-M. Markers below the amino acid sequence represent: *, identity; : , strongly similarity; ., weakly similarity. Amino acid residues shown by white letters on black basis present citrate allosteric binding site [12]. Amino acid residues at the C-terminus extension marked with grey background represent the motif responsible for inhibition by ATP [13]. (TIF) [file pone.0019645.s001.tif]

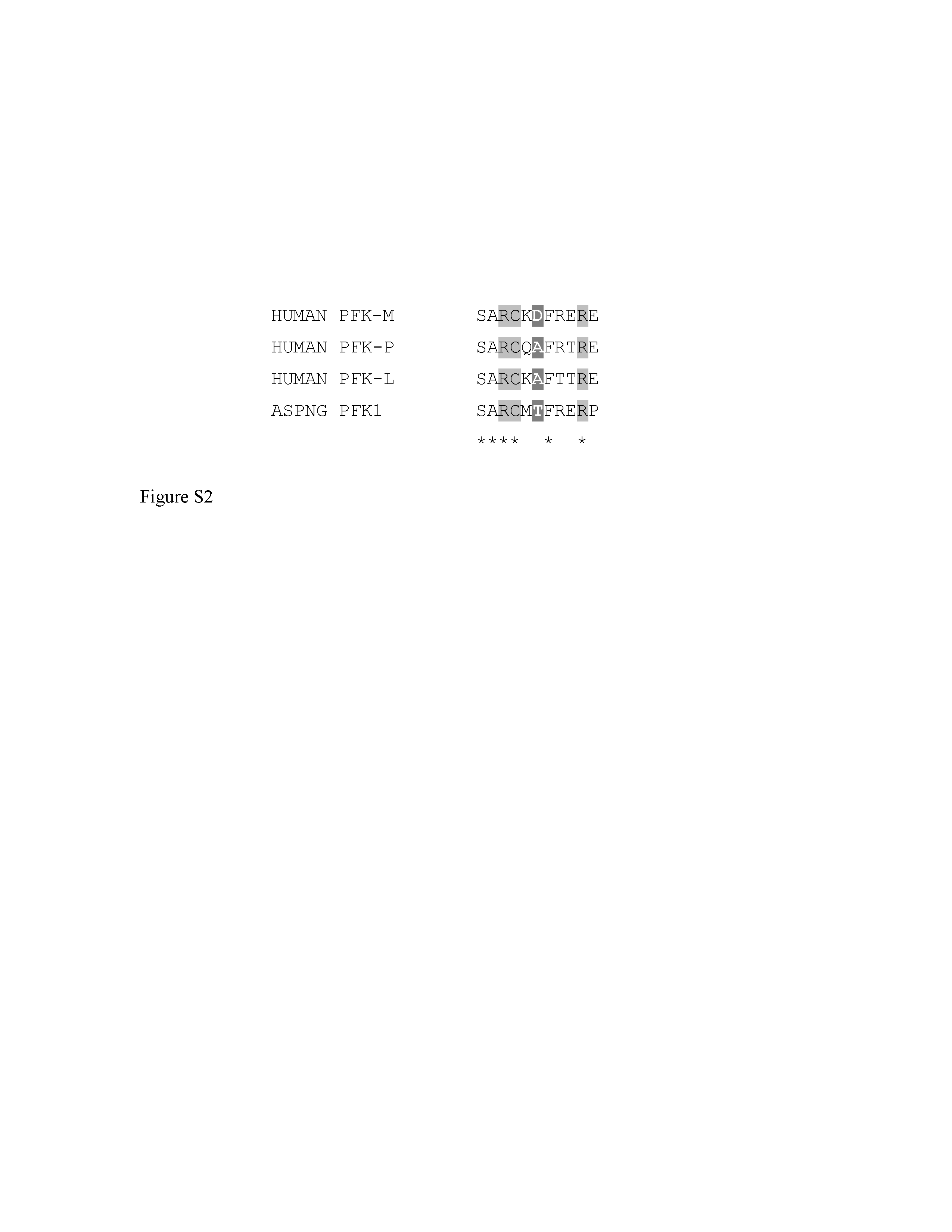

Supplement: Figure S2 — Alignment of the deduced amino acid residues of the PFK1 active center of human PFK1 proteins PFK-M (P08237), PFK-P (Q01813), PFK-L (P17858), and A. niger (P78985) are shown. The threonine (T) residue, located in the enzyme active center, must be phosphorylated in order to regain activity of the shorter A. niger PFK1 fragmen [14]. Only the aspartate residue (D) of PFK-M exhibits a negative charge similar to the phosphorylated threonine in the A. niger fragment, unlike the alanine residues of the PFK-P and PFK-L human isoforms (while letters). Allosteric binding sites for ATP are marked with a gray background. (TIF) [file pone.0019645.s002.tif]

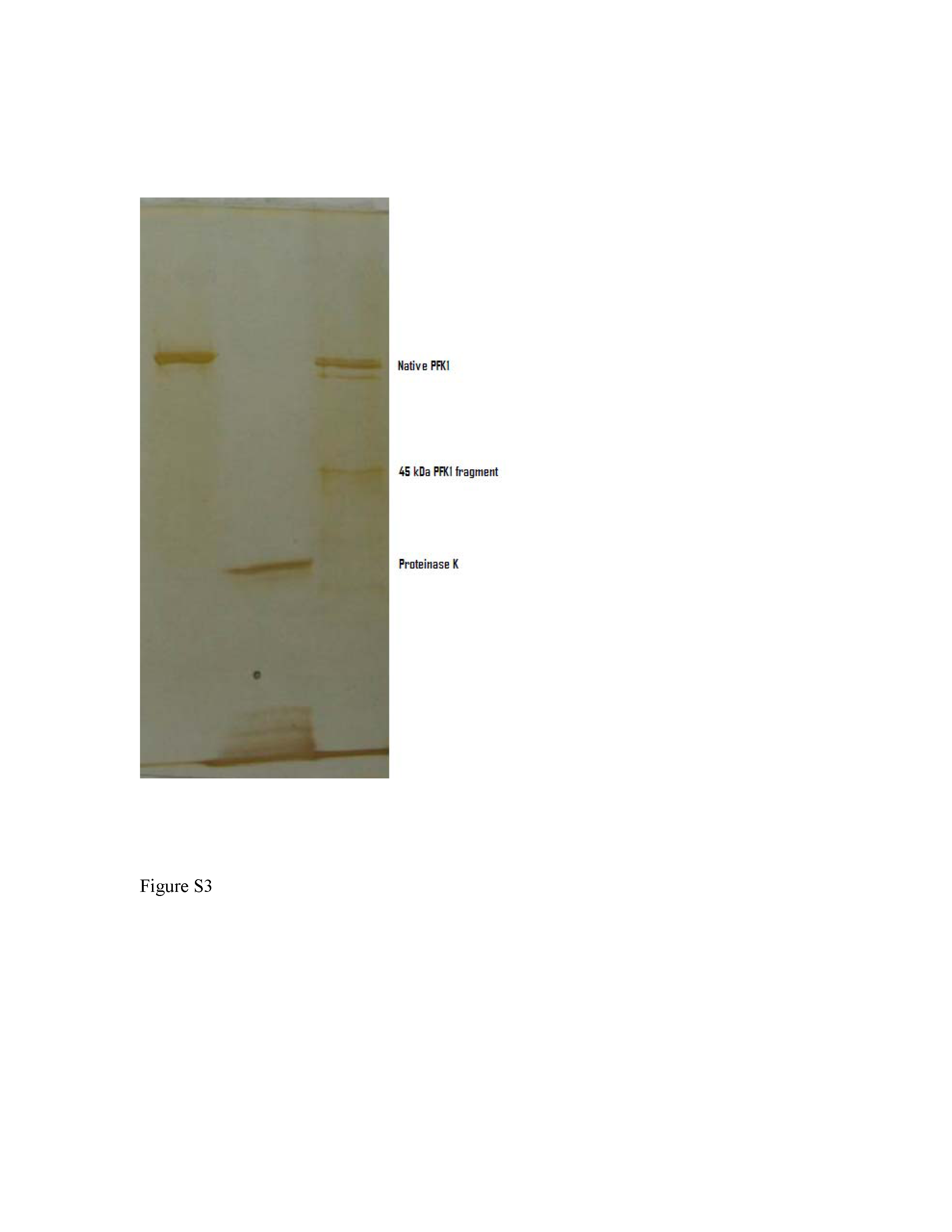

Supplement: Figure S3 — SDS-PAGE of the native PFK1 after limited proteolytic degradation by Proteinase K. SDS-PAGE of the native PFK1 isolated from rabbit muscle revealed the formation of a 45-kDa fragment after limited proteolysis by Proteinase K. From left to right: purified native PFK1 from rabbit muscle; Proteinase K; native PFK1 after limited proteolysis with Proteinase K (0,001 mg/ml). (TIF) [file pone.0019645.s003.tif]

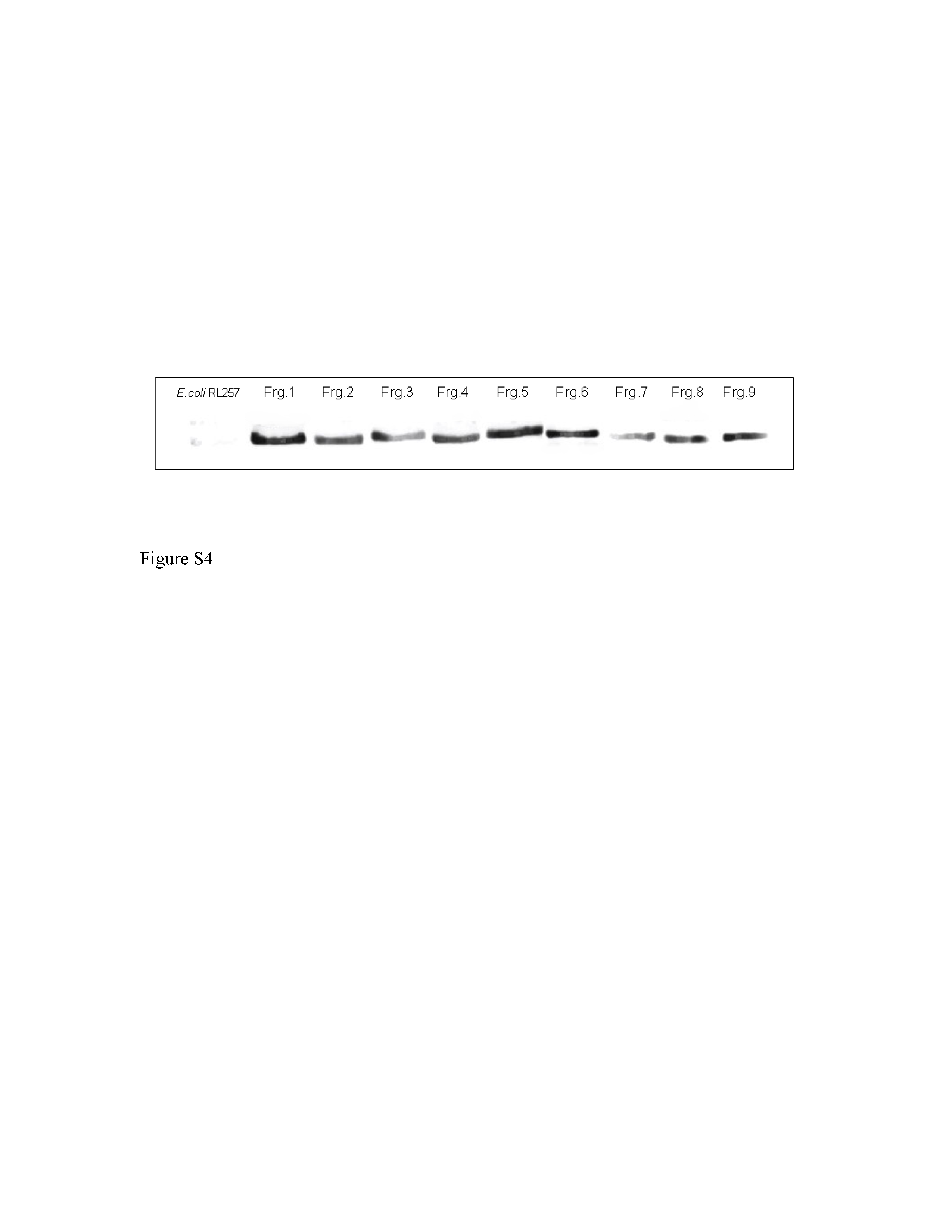

Supplement: Figure S4 — Western blot of PFK-M fragments synthesized in E.coli . Fragments of various length were encoded by a series of truncated pfkM genes in E.coli strain RL257 lacking its own PFK1 enzymes. (TIF) [file pone.0019645.s004.tif]

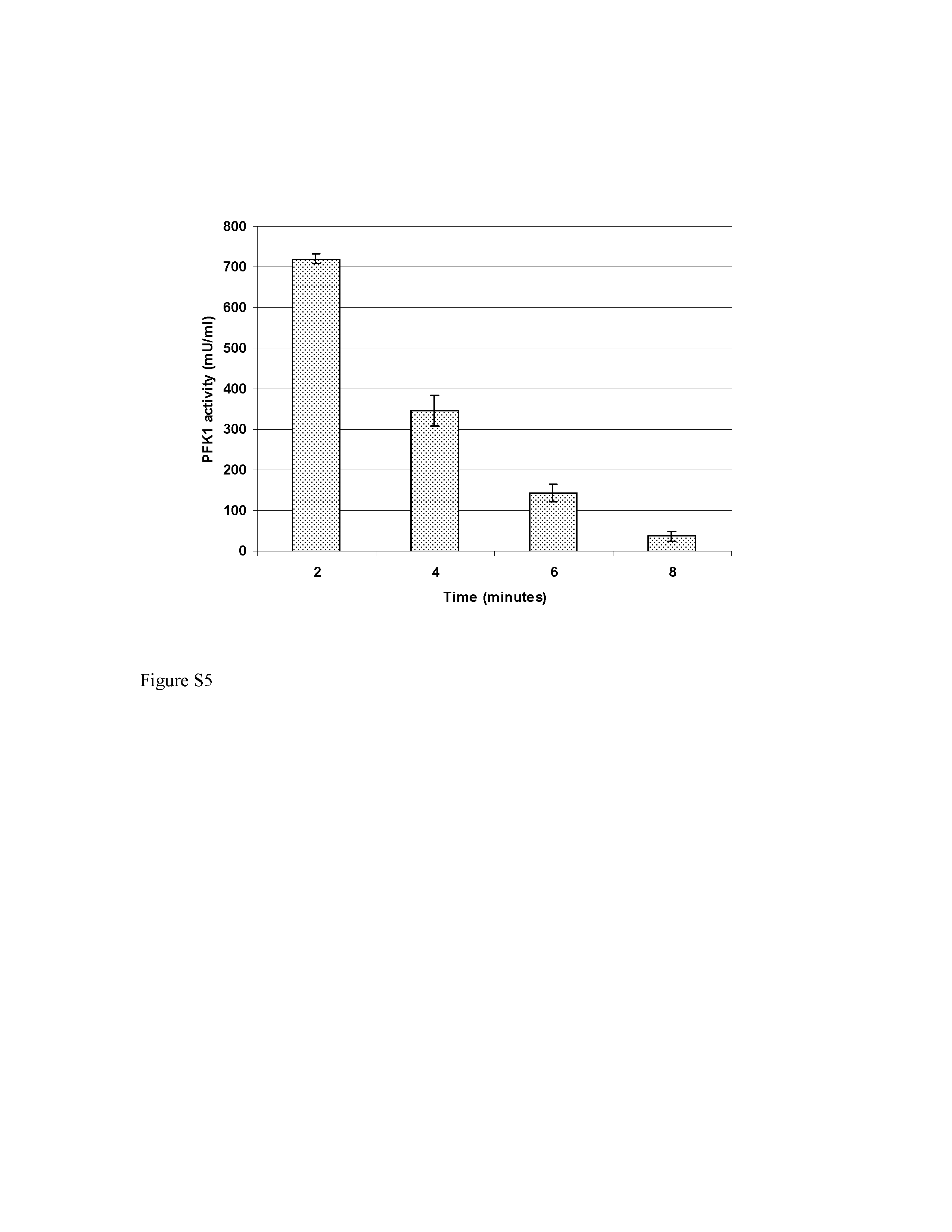

Supplement: Figure S5 — Instability of the shorter PFK-M fragment under in vitro conditions. PFK1 activity was rapidly lost in the measuring system with low protein concentration. Data are presented as means ± standard deviation. (TIF) [file pone.0019645.s005.tif]
