# Supplementary material for: Posttranslational Modification of 6-phosphofructo-1-kinase as an Important Feature of Cancer Metabolism
Source: PLoS One. 2011 May 4;6(5):e19645. doi: 10.1371/journal.pone.0019645 (PMC3087806; doi:10.1371/journal.pone.0019645)
Supplement: Text S1 — Supplementary data (DOC) [file pone.0019645.s008.doc]

**Supplementary data**

**Biological materials**

***Cell lines***

HeLa, a human cervical adenocarcinoma cell line (1), B16-F10, a mouse melanoma cell line (2), and human embryonic kidney (HEK 293) cells (3) were grown in DMEM (Invitrogen, Carlsbad, CA) medium with 10% FBS (Invitrogen, Carlsbad, CA). Adhered cells were released from the surface by Trypsin/EDTA (Invitrogen, Carlsbad, CA). Cell-free extraction was performed in RIPA extraction buffer (Pierce, Rockford, IL) containing 1 mM dithiothreitol (DTT), 1μL protease inhibitor cocktail and 1 mM phenylmethylsulfonyl fluoride (PMSF).

TF-1, a human erythroleukemic cell line (4) and Nb2-11, a rat lymphoma cell line (5), were grown in RPMI-1640 (Invitrogen, Carlsbad, CA) medium with 10% FBS. After the cells were collected by centrifugation, total proteins were extracted in RIPA buffer containing 1 mM DTT, protease inhibitor cocktail and 1 mM PMSF.

***Tissue specimens***

Both flanks on C57BL/6 mouse were shaved and subcutaneously injected with B16-F10 melanoma cells. After 10 days, the animal was euthanized and the entire tumor mass dissected excluding any extraneously host tissue. Isolated tumor was frozen under the liquid nitrogen and later stored at -80^o^C until analysed. Frozen tumor cells were finally disrupted with Mikro-Dismembrator S (Sartorius, BBI Systems, Germany) and total proteins extracted in RIPA buffer containing 1 mM DTT, protease inhibitor cocktail and 1 mM PMSF.

Cell-free extracts of soluble proteins were used for SDS-PAGE.

***Lymphocyte isolation***

Peripheral blood mononuclear cells that mainly contained T and B lymphocytes were isolated by the Histopaque/Ficoll method. After density gradient separation by centrifugation, mononuclear cells were collected from the plasma/Ficoll interface. Proteins were extracted in RIPA buffer (Pierce) containing 1 mM DTT and 1mM PMSF.

***E.coli***

*E.coli* cells (strain RL257) (lac^+^, *lac*I^q^, ∆(*arg*F-*lac*)U169, *ara*D139, *rsp*L150, *pts*F25, ∆*pfk*B::*kan*, *flh*D5301, ∆*pfk*A::*kan*, *rps*R, *deo*C, *rel*A1) with disrupted native bacterial *pfk*A and *pfk*B (6) were used for testing truncated human *pfk*M genes. For the amplification of plasmid pALTER-Ex1 (Promega, Southampton, UK), the *E.coli* JM109 strain was used. Transformants were propagated on LB medium with tetracycline (10 μl/ml).

***HEK 293 cell line***

For the construction of isogenic stable cell lines, the Flp-In T-Rex-HEK293 cell line (Invitrogen, Carlsbad, CA) was utilized.

**Purification of PFK1 from rabbit muscle**

Rabbit skeletal muscle PFK1 was prepared from fresh muscle. The tissue was first frozen in liquid nitrogen and ground by a Mikro-Dismembrator S (Sartorius, BBI Systems, Germany). Soluble proteins were extracted with 100 mM phosphate buffer pH 7.8 containing 0.15 mM glycerol, 1 mM DTT, 0.5 mM EDTA, and 1 mM PMSF. The same buffer was used throughout all purification steps. PFK1 was precipitated by ammonium sulfate in the range of 35-55% saturation. After re-dissolving and desalting the homogenate (Sephadex^TM^ G-25M columns), proteins were separated by gel filtration using a Sepharose 12 column (Pharmacia, Uppsala, Sweden). The fraction showing PFK1 activity was then applied to an Aminophenyl-ATP Sepharose affinity column (Jena Bioscience, Jena, Germany) and eluted with 6 mM fructose-6-phosphate and 1 mM ADP. The enzyme was concentrated to more than 5 mg/ml using an ultrafiltration device (Amicon Millipore, Billerica, MA). Glycerol was added to a final concentration of 20% (v/v) and samples were stored at -20°C.

The homogeneity of the protein sample and the presence of shorter fragments were verified by SDS-PAGE (7) in a minigel system (LKB, Bromma, Sweden). Molecular mass markers MW-SDS-70L (Sigma-Aldrich, Steinheim, Germany) were used as standards.

Identical procedure was used to isolate the recombinant human native PFK-M from the *E.coli* RL257 transformant.

Total protein concentration of the samples was determined by bicinchoninic acid protein assays (8) performed with the Sigma-Aldrich kit (Steinheim, Germany).

**Testing *E. coli* transformants for growth on glucose**

Transformed *E.coli* cells (strain RL257) were propagated on LB medium with tetracycline (10 μl/ml) by incubating on a rotary shaker at 37°C overnight. One hundred milliliters of supplemented glucose medium (5% glucose, 0.2% Amicase (Sigma-Aldrich, Steinheim, Germany), M9 salts) with tetracycline (10 μl/ml) and 1 mM iso-propyl-thio-galactoside (IPTG) was inoculated with 100 μl of the cells from the LB culture and incubated on a rotary shaker at 37°C. Aliquots of the culture were removed at the indicated times and the optical density at 600 nm was measured.

**Cell-free extracts of transformed *E.coli* cells**

To detect PFK-M activities in the homogenates of *E.coli* transformants, cells were grown in 100 ml of LB medium on a rotary shaker at 37°C. In the exponential growth phase (OD 0.5), IPTG was added at a final concentration of 1 mM and the incubation continued at 30°C for 4 hours. The cells were harvested by centrifugation at 5000 rcf for 5 minutes and washed by centrifugation using ice-cold extraction buffer. The cell pellet was frozen with liquid nitrogen and ground in the Mikro-Dismembrator (Sartorius AG, Gottingen, Germany). Proteins from the crushed cells were extracted with cold 50 mM HEPES buffer pH 7.5 containing 100 mM KCl, 5 mM MgSO_4_, 10% v/v polyethylene glycol (PEG 6000), 1 mM DTT, 1 mM PMSF, and 0.5 mM EDTA. Normally the total protein concentration in the cell free homogenate was higher then 10 mg/L.

Fructose-6-phosphate was added to the homogenate at a final concentration of 6 mM before the cell debris was removed by centrifuging at 12,000 rcf for 10 minutes. PEG 6000 (9) and fructose-6-phosphate (10) were reported to extenuate the dissociation of the PFK1 tetrameric structure and thus prevent deactivation of the protein. Throughout all the steps, the homogenate was maintained at ≤ 4°C.

**Enzyme assays**

PFK1 activities were measured spectrophotometrically at 340 nm (Lambda25 UV/VIS spectrophotometer, Perkin Elmer) using a coupled reaction system, essentially as reported previously (11). Unless otherwise stated, the assay mixture contained, in a final volume of 1 mL: 50 mM HEPES buffer (pH 7.5), 10% v/v PEG 6000, 1 mM DTE, 100 mM KCl, 5 mM MgCl_2_, 0.2 mM NADH, specified concentrations of F6P and ATP, 0.9 U/mL aldolase (Sigma-Aldrich, Steinheim, Germany), 15 U/mL triosephosphate isomerase and 15 U/mL glycerol-3-phosphate dehydrogenase (Sigma-Aldrich, Steinheim, Germany) and 20 μL of cell free homogenate. Before use, the auxiliary enzymes were dialyzed overnight at 4°C against 50 mM HEPES buffer (pH 7.5) containing 1 mM DTE, with one change of buffer after 8 h. Due to the extreme instability of the shorter PFK1 fragments (supplemental Fig. S4), enzyme activities in eight individual vials were measured simultaneously, using Cell Changer System (Perkin Elmer). The enzyme reactions were started by adding homogenate aliquots into the individual vials. All presented kinetic data are averages obtained from a minimum of three replicate measurements.

**Growth of transfected cells HEK 293 cells**

Cell numbers were determined with the 3-(4,5-dimethylthiazolyl-2)-2,5-diphenyltetrazolium bromide (MTT) assay (ATCC, Manassas, Virginia). Cells were plated into 96-well plates at initial concentrations of 1 × 10^3^ and 1 × 10^4^ cells per well in a final medium volume of 180 μl. For each final result, the cell numbers from 5 parallel wells were determined. Growth was followed for four days and one 96-well plate was used for each day. Before the measurement, the used medium was removed and stored at -20°C until the time of glucose and lactate determination. Fresh medium with MTT at a final concentration of 0.5 mg/ml was added to the cells attached to the bottom of each well and incubated for 4 hours. After that, the supernatant was removed and 200 μl of DMSO was added. Finally, the optical density was measured using a micro plate reader (Power Wave XS, Bio-Tek Instruments, Inc., Winooski, VT) at 570 nm. Cell numbers per wells were determined from a calibration curve.

**Glucose and lactate measurements**

In parallel to cell concentration measurements, glucose consumption and lactate formation were determined in the medium. The levels of both metabolites were measured using enzymatic kits according to manufacturer’s instructions (Megazyme Int., Wicklow, Ireland).

**References:**

1. Scherer WF, Syverton JT, Gey GO (1953) Studies on the propagation in vitro of poliomyelitis viruses. IV. Viral multiplication in a stable strain of human malignant epithelial cells (strain HeLa) derived from an epidermoid carcinoma of the cervix. J Exp Med 97(5): 695-710.

2. Fidler IJ (1975) Biological behavior of malignant melanoma cells correlated to their survival in vivo. Cancer Res 35(1): 218-224.

3. Graham FL, Smiley J, Russell WC, Nairn R (1977) Characteristics of a human cell line transformed by DNA from human adenovirus type 5. J Gen Virol 36(1): 59-74.

4. Kitamura T, Tojo A, Kuwaki T, Chiba S, Miyazono K et al. (1989) Identification and analysis of human erythropoietin receptors on a factor-dependent cell line, TF-1. Blood 73(2): 375-380.

5. Gout PW, Horsman DE, Fox K, De Jong G, Ma S et al. (1994) The rat Nb2 lymphoma: a novel model for tumor progression. Anticancer Res 14(6B): 2485-2492.

6. Lovingshimer MR, Siegele D, Reinhart GD (2006) Construction of an inducible, pfkA and pfkB deficient strain of Escherichia coli for the expression and purification of phosphofructokinase from bacterial sources. Protein Expr Purif 46(2): 475-482.

7. Laemmli UK (1970) Cleavage of structural proteins during the assembly of the head of bacteriophage T4. Nature 227(5259): 680-685.

8. Smith PK, Krohn RI, Hermanson GT, Mallia AK, Gartner FH et al. (1985) Measurement of protein using bicinchoninic acid. Anal Biochem 150(1): 76-85.

9. Reinhart GD (1980) Influence of polyethylene glycols on the kinetics of rat liver phosphofructokinase. J Biol Chem 255(22): 10576-10578.

10. Reinhart GD, Lardy HA (1980) Rat liver phosphofructokinase: kinetic and physiological ramifications of the aggregation behavior. Biochemistry 19(7): 1491-1495.

11. Usenik A, Legiša M (2010) Evolution of allosteric binding sites on 6-phosphofructo-1-kinase. PLoS One 5(1): e15447.

12. Kemp RG, Gunasekera D (2002) Evolution of the allosteric ligand sites of mammalian phosphofructo-1-kinase. Biochemistry 41(30): 9426-9430.

13. Martinez-Costa OH, Hermida C, Sanchez-Martinez C, Santamaria B, Aragon JJ (2004) Identification of C-terminal motifs responsible for transmission of inhibition by ATP of mammalian phosphofructokinase, and their contribution to other allosteric effects. Biochem J 377: 77-84.

14. Capuder M, Šolar T, Benčina M, Legiša M (2009) Highly active, citrate inhibition resistant form of *Aspergillus niger* 6-phosphofructo-1-kinase encoded by a modified *pfk*A gene. J Biotechnol 144(1): 51-57.
